# Federated parameter-free DBSCAN clustering and its application in image recognition

**DOI:** 10.1371/journal.pone.0355161

**Published:** 2026-08-04

**Authors:** Fang Cheng, Zilong Deng, Mustafa Muwafak Alobaedy, Xiaocun Huang

**Affiliations:** 1 College of Information Technology, Anqing Vocational and Technical College, Anqing, China; 2 Faculty of Information Technology, City University Malaysia, Petaling Jaya, Malaysia; 3 Cangzhou Normal University, Cangzhou, China; University of Lagos Faculty of Engineering, NIGERIA

## Abstract

DBSCAN (A Density-Based Algorithm for Discovering Clusters in Spatial Databases with Noise) is a classic clustering algorithm. However, clustering distributed data with privacy protection in edge computing environments is a key challenge for DBSCAN. In this research, we combine federated clustering and DBSCAN and propose two secure federated parameter-free DBSCAN clustering methods, called FDBSCAN and FDBSCAN++. The process involves the following steps: (1) differential privacy is applied to the client data and adaptive DBSCAN is used at each client to identify core points; (2) the clients send the extracted core points to the server, where the server aggregates these to obtain the final global cluster centers (FDBSCAN and FDBSCAN++ use different methods in this step); (3) the final clusters are generated using these global centers. To verify the effectiveness of the proposed two algorithms, we use eight real datasets, including the large-scale image dataset MNIST. Compared with traditional and state-of-the-art (SOTA) improved DBSCAN and federated clustering algorithms, the proposed algorithms achieve better clustering accuracy. In addition, we also apply FDBSCAN++ to image clustering and segmentation tasks, which achieves satisfactory results.

## 1. Introduction

DBSCAN is a classic density-based spatial clustering algorithm, which can partition areas of sufficiently high density into clusters and discover arbitrarily shaped clusters in spatial databases with noise [1]. DBSCAN is a powerful tool that is applied in many applications, such as industrial applications [2], analysis of ship traffic behavior [3], market analysis [4], medical data processing [5], urban construction planning [6], network security, remote sensing, *etc.* Many scholars have proposed different improved algorithms, such as parameter optimization, identification and processing of clusters with different densities, big data processing, high-dimensional data processing, and optimization for specific application scenarios.

With the rapid development of edge intelligence and mobile Internet, clustering algorithms have encountered new problems: how to perform clustering distributed data under the condition of privacy protection? DBSCAN also faces this problem, which is the motivation of our research. We try to make DBSCAN clustering for distributed data under privacy requirements.

**Fig 1:**
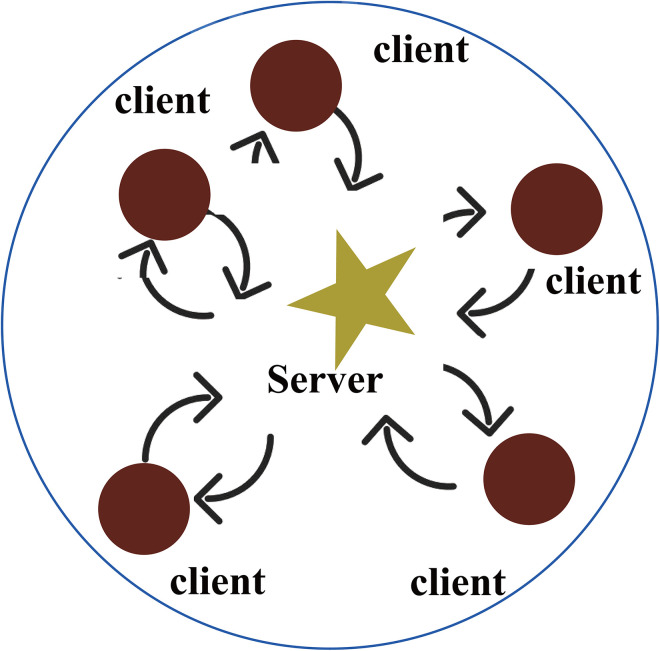
The framework of secure federated clustering: All clients collaboratively generate the final clustering results without leaking the original data.

An emerging clustering framework provides the answer, namely secure federated clustering as shown in Fig 1: Each client uploads the desensitized data to the server, and the server sends the processed results to each client. Each client forms the final clusters based on the received results [7,8].

Based on this idea, we propose two kinds of secure federated parameter-free DBSCAN clustering, namely FDBSCAN and FDBSCAN++, with two different global cluster center generation strategies. Specifically, in the client, we use differential privacy to encrypt the raw data, ensuring that it will not be leaked in subsequent calculations. Based on the encrypted data, we construct a parameter free DBSCAN clustering algorithm, which can be run by any data party without adjusting parameters. Then, the client uploads the core points obtained from DBSCAN to the server. The server aggregates all the core points and uses two different methods to obtain the global class center, one is local density calculation (in FDBSCAN) and the other is k-means clustering algorithm (in FDBSCAN++). Finally, the server sends the global class to each client, and the client assigns the local raw data to the nearest global class center to form the final clustering results. The main contributions of this research are as follows:

- We propose the first secure federated DBSCAN clustering framework and achieved satisfactory clustering results.
- We propose an adaptive DBSCAN clustering algorithm that utilizes other clustering algorithms to construct pseudo labels, helping DBSCAN find better parameter values.

The rest of this paper is organized as follows. We report the related work on DBSCAN in Section 2. We introduce the proposed method FDBSCAN in Section 3, and FDBSCAN++ in Section 4. In Section 5, we perform extensive experiments to compare the proposed methods with thirteen benchmark models and discuss the results. Finally, in Section 6, we conclude our paper and explore future work.

## 2. Related work

DBSCAN has two parameters: *Eps*: The maximum distance between two points for them to be considered neighbors. *minPts*: The minimum number of points required to form a dense region (cluster). DBSCAN defines three kinds of data points: A point is a core point if it has at least minPts points within its *Eps*-neighborhood (including itself). A border point has fewer than minPts within *Eps* but is reachable from a core point. A noise point is neither a core nor a border point. Algorithm Process is as follows: Step 1: Start with an arbitrary unvisited point. Step 2: Retrieve all points within its *Eps*-neighborhood. If it is larger than minPts neighbors, form a new cluster. If not, mark it as noise (may be reassigned later as a border point). Step 3: For each core point, expand the cluster: Add all directly reachable points (within *Eps*) to the cluster. Recursively process new core points to include their neighbors (density-connected points). Step 4: Repeat until all points are visited (classified into clusters or marked as noise).

Most algorithms improve DBSCAN from the following five perspectives:

(1) Parameter optimization. Zhang *et al.* leveraged the whale optimization algorithm’s global search capability and rapid convergence properties to automatically determine optimal parameters, thereby reducing parameter sensitivity [9]. Regarding the issue of difficult automatic determination of parameters, Starczewski *et al.* automatically determine parameters based on data features, which reduces user intervention in parameter selection [10]. Zhang *et al.* proposed the automated DBSCAN parameter search framework DRL-DBSCAN guided by deep reinforcement learning to reduce dependence on expert knowledge. Based on a small number of external clusters indices, rewards were constructed to enable DRL agents to learn the optimal parameter search strategy that adapts to different data distributions [11].
(2) Identify clusters with different densities. Hu *et al.* improved DBSCAN based on hybrid algorithms, cleverly utilizing reverse nearest neighbors and influence space to identify core objects, thereby achieving effective processing of clustering with different densities [12].
(3) Clustering big data. Hanafi *et al.* proposed a fast DBSCAN algorithm based on reducing computational complexity, which uses local datasets (operational datasets) to reduce computational complexity and reduces distance calculations by limiting the search space [13]. Weng *et al.* improved the h-DBSCAN algorithm based on hierarchical search technology, using hierarchical navigable small world (HNSW) technology to optimize search efficiency and reduce unnecessary *Eps*-neighborhood queries [14].
(4) Clustering high-dimensional data. Braune *et al.* proposed black hole clustering and protoclustering methods based on reducing parameter adjustments and optimizing high-dimensional data. These methods aim to reduce the need for parameter adjustments while optimizing the clustering effect of high-dimensional data [15]. Li *et al.* proposed an improved DBSCAN algorithm based on neighborhood similarity and retrieval efficiency considerations. It utilizes neighborhood similarity to reduce redundant distance calculations and uses a cover tree to improve retrieval efficiency, avoiding treating all dimensions equally [16].
(5) Specific application scenario optimization. Jin *et al.* proposed a variable scale HCA-DBSCAN method for detecting anomalies in multidimensional energy data in the steel industry [2]. Wei *et al.* proposed an algorithm that combines multidimensional dynamic time warping (MDDTW) and adaptive DBSCAN based on the demand for pattern recognition of marine traffic behavior [3].

Unlike previous work, in this research, we focus on a new problem of DBSCAN: how to clustering distributed data under privacy requirements? To address this issue, we propose a federated DBSCAN. In addition, we propose an adaptive DBSCAN to avoid human intervention at the clients.

## 3. FDBSCAN: A federated parameter-free DBSCAN clustering algorithm

FDBSCAN consists of three steps: (1) protect local data of clients by using differential privacy, and use adaptive DBSCAN to get core points at the clients; (2) obtain the final global centers at the server; (3) final clusters generation.

### 3.1. Protect local data of client by using differential privacy

Differential privacy is a data protection method with a rigorous mathematical foundation [17]. The main definitions are as follows:

**Definition 1:** ϵ**-Differential privacy.** For a random algorithm *M* and two adjacent datasets *D* and $D^{\prime }$ with only one different record [18], the algorithm *M* meets differential privacy if it satisfies the following formula:


$$\begin{array}{c} \frac{Pr[M(D)\in S]}{Pr[M(D^{\prime })\in S]}\leq e^{\epsilon }, \end{array}$$ (1)

where Pr[...] denotes the probability of an event, *S* denotes the results of *M*, and ϵ denotes the privacy budget.

**Definition 2: Global sensitivity.** For a query function *f*() and two adjacent datasets *D* and $D^{\prime }$, global sensitivity Δf is defined as $\Delta f=\max_{D,D^{\prime }}\|f(D)-f(D^{\prime }){\|}_{1}$, where $\|...{\|}_{1}$ denotes the L1-norm, Δf denotes the difference degree in query results between two datasets *D* and $D^{\prime }$. Actually, in this research, *f*() denotes a clustering algorithm, Δf=1 because the difference of clustering *D* and $D^{\prime }$ equals 1.

**Definition 3: Laplacian mechanism.** *M*(*D*) is the function with Laplacian noise, then M(D)=f(D)+Lap(Δf/ϵ,μ), where Lap(Δf/ϵ,μ) denotes random Laplacian noises generated by Laplacian distribution: $Lap(\lambda ,\mu )=\frac{1}{2\lambda }e^{-(x-\mu )/\lambda }$.

In practical applications, the value of ϵ is set randomly, and the value of μ is set to zero. The main implementation mechanism of differential privacy is to add Laplacian noise to the data, and in this paper, we also take the same operation.

The specific approach is as follows: Based on previous definitions, we can use Laplacian noises Lap(Δf/ϵ) to protect raw data in each client $c_{i}$ (totally num_clients clients), global sensitivity Δf=1 in most clustering algorithm, and we also set it to 1 in FDBSCAN. Specifically, For local data $d^{i}$ in client $c_{i}$, we add Laplacian noises Lap(1/ϵ) to $d^{i}$, and the privacy data $p_{i}$ is defined as:


$$\begin{array}{c} p_{i}=d^{i}+Lap(1/\epsilon ). \end{array}$$ (2)

Subsequent calculations only utilize private data rather than raw data. In the process of data exchange between the client and server, even if the exchanged information is leaked, it will not result in the leakage of raw data.

### 3.2. Adaptive DBSCAN at the clients

Neural networks use labels and prediction results to construct an objective function that solves for the most parameterized set (weights) in order to achieve better classification accuracy. Taking inspiration from this, we construct a neural network-like objective function to solve for better DBSCAN parameters: *Eps* and *minPts* as follows:


$$\begin{array}{c} \underset{\theta \in f()}{\text{arg}\max}g(f(),\pi ), \end{array}$$ (3)

where *g*() denotes the objective function, *f*() denotes clustering results generated by DBSCAN, π denotes pseudo label, and θ denotes parameter *Eps* and *minPts*.

When the amount of shared information between clustering results and pseudo labels is greater, the clustering results may be better, and we can obtain a better set of parameters. Based on the above ideas, we use the Adjusted Rand Index (ARI) as a tool to measure the amount of shared information as follows:


ARI=a−1(n2)bc(b+c)/2−1(n2)bc, (4)

a=∑i=1r∑j=1t(nij2), (5)

b=∑i=1r(ni.2), (6)

c=∑j=1t(n.j2), (7)

where all the datasets are divided into *r* clusters according to ground-truth labels and *t* clusters according to clustering algorithm, *n* denotes the number of data points, and (n2)=n(n−1)/2.

To explain this equation, we give an example, there are six samples, according to the label, they are divided into two clusters: A (1,2,3) and B (4,5,6), according to the clustering results, they are divided into two clusters: C (1,2,4) and D (3,5,6). Any two data points $x_{i}$ and $x_{j}$ form a sample pair, the number of sample pairs is n(n−1)/2. Based on (*A*,*B*) and (*C*,*D*), we can construct a contingency table. $n_{A,C}=|\{1,2,3\}\cap \{1,2,4\}|=|\{1,2\}|=2$, similarly, $n_{A,D}=1$, $n_{B,C}=1$, $n_{B,D}=2$. Then, a=∑i=1r∑j=1t(nij2)=(22)+(12)+(12)+(22)=1+0+0+1=2. b=∑i=1r(ni.2)=6, c=∑j=1t(n.j2)=6, (n2)=15. Thus, $ARI=\frac{2-36/15}{6-36/15}\approx -0.11.$

Another issue is how to obtain pseudo-labels. We use the k-means clustering algorithm to generate pseudo labels. K-means has a parameter, *i.e.,* the number of clusters, we set it to $\lceil \sqrt{N}\rceil$. Thus, we can obtain the final objective function:


$$\begin{array}{c} \underset{\text{Eps},\text{minPts}}{\text{arg}\max}ARI(\text{DBSCAN}(\text{Eps},\text{minPts}),\pi |\text{k-means}). \end{array}$$ (8)

To solve this objective function, we take two steps: (1) optimizing parameter *Eps* while fixing parameter *minPts*, (2) optimizing parameter *minPts* while fixing parameter *Eps*.

We first optimize parameter *minPts* while fixing parameter *Eps*, the best value of *minPts* (minPts_b) can be obtained by


$$\begin{array}{c} \text{minPts\_b}=\underset{\text{minPts}}{\text{arg}\max}ARI(\text{DBSCAN}|\text{Eps\_b},\pi ), \end{array}$$ (9)

then, we optimize parameter *Eps* while fixing parameter *minPts*, the best value of *Eps* (Eps_b) can be obtained by


$$\begin{array}{c} \text{Eps\_b}=\underset{\text{Eps}}{\text{arg}\max}ARI(\text{DBSCAN}|\text{minPts},\pi ). \end{array}$$ (10)

By using the above two formulas, the optimal *Eps* and *minPts* can be iteratively solved. The specific solving process is shown in the algorithm 1.

**Algorithm 1: the process of solving Formula (8).**



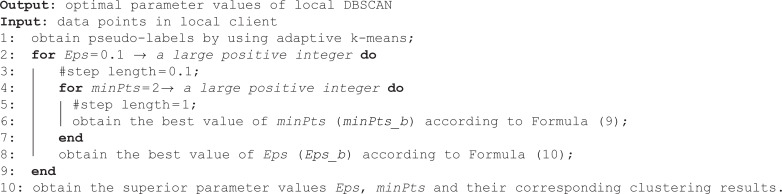



After obtaining superior values of parameter *Eps*, *minPts*, we can get the core points (*cp*) by DBSCAN(*Eps_b*,*minPts_b*), core points are defined by DBSCAN. Each client uploads core points to the server.

### 3.3. Obtain the final global centers at the server

The server aggregates the core points from all the clients (*CP*), and we use the following method to obtain the final global centers (*center*) as follows:

For a dataset $\text{D}=\{x_{1},x_{2},...,x_{n}\}$, $x_{i}=\{x_{i}^{1},x_{i}^{2},...,x_{i}^{d}\}$, the SNN of any two data points $x_{i}$ and $x_{j}$ is defined as follows: $\text{SNN}(x_{i},x_{j})=\text{KNN}(x_{i})\cap \text{KNN}(x_{j}),$ where $\text{KNN}(x_{i})$ and $\text{KNN}(x_{j})$ denote *k* nearest neighbors of $x_{i}$ and $x_{j}$, respectively. If the value of $\text{SNN}(x_{i},x_{j})$ is bigger, it means $x_{i}$ and $x_{j}$ have more common neighbors. We use *sim* to describe the structural similarity between two data points $x_{i}$ and $x_{j}$. The $\text{sim}(x_{i},x_{j})$ is defined as follows:


$$\begin{array}{c} \text{sim}(x_{i},x_{j})=\left\{ \begin{array}{cc} \frac{|\text{SNN}(x_{i},x_{j}){|}^{2}}{\sum_{p\in \text{SNN}(x_{i},x_{j})}(d_{ip}+d_{jp})}, & i,j\in \text{SNN}(i,j),\\ 0, & \text{otherwise}, \end{array}\right. \end{array}$$ (11)

where $d_{ip}$ and $d_{jp}$ are Euclidean distance.

The local density of $x_{i}$ is defined as follows: $\rho_{i}=\sum_{x_{j}\in \text{KNN}(x_{i})}\text{sim}(x_{i},x_{j})$, the related distance of data point $x_{i}$ is defined as follows: $\delta_{i}=\min_{x_{j}:\rho_{p_{j}}>\rho_{x_{i}}}[d_{ij}(\sum_{x_{p}\in \text{KNN}(x_{i})}d_{ip}+\sum_{x_{q}\in \text{KNN}(x_{j})}d_{jq})].$ We use a variable γ to select cluster centers as follows: $\gamma_{i}=\rho_{i}*\delta_{i}$, where $\rho_{i}$ and $\delta_{i}$ denotes the local density and relative distance of data point *i*, respectively. Then, the top *k* data points with large γ values are selected as final centers.

### 3.4. Final clusters generation

After obtaining the global cluster center, all the clients assign the raw data to the nearest global cluster center to form the final clusters. The whole process of FDBSCAN is shown in Algorithm 2.

**Algorithm 2: The proposed FDBSCAN algorithm**



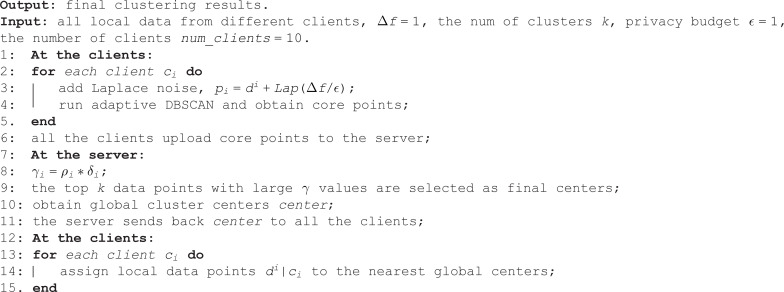



### 3.5. Remarks about the proposed FDBSCAN

**Time Complexity:** See the Algorithm 2, for line 3, adding Laplace noises has *O*(*n*) time. For line 4, adaptive DBSCAN needs *O*(*n*^2^) time, see the Algorithm 1, we use a double for loop to automatically find the optimal parameter values, but the number of times each loop is executed is limited. Generally speaking, we often set Eps∈{0.1,5} (step length is 0.1), minPts∈{2,5} (step length is 1), the Algorithm 1 has $O(p*n^{2})$, where *p* is a constant. Thus, in the Algorithm 2, lines 1–5 have *O*(*n*^2^) time. Lines 7–11 have *O*(*n*^2^) time, because $\rho_{i}$ and $\delta_{i}$ are based on $\text{SNN}(x_{i},x_{j})$ which has *O*(*n*^2^) time. Lines 12–15 have *O*(*n*) time because each data point is visited only once. In short, the time complexity of FDBSCAN is $O(n^{2}+n^{2}+n)\approx O(n^{2})$.

**Privacy–utility Trade-offs:** We utilize differential privacy to safeguard the client’s raw data. Differential privacy is a prevalent privacy protection technique frequently employed in the protection of clustering algorithms, as proved in our prior work [7,8]. The specific implementation method is to add Laplacian noise to the original data as shown in Equation (2), we use encrypted raw data to calculate core points, and upload the core points to the server, no client can infer the raw data of other clients through uploaded core points even if the server is untrustworthy.

In the proposed algorithm, we first encrypt local data via differential privacy. Within the range of ϵ∈[0,1], the privacy budget can achieve favorable privacy protection performance. Specifically, the optimal clustering performance is obtained when the privacy budget ϵ is set to 1.

Our work adopts Laplacian mechanism for differential privacy, where the perturbed data is defined as $\rho_{i}=d^{i}+\text{Lap}(1/\epsilon )$ with global sensitivity Δf=1. There exists an inherent trade-off between privacy protection and clustering utility controlled by ϵ:

- When ϵ→0: Large Laplacian noise severely distorts original data. Although privacy leakage risk is minimized, the extracted core points deviate far from real data density distribution, resulting in poor ARI and NMI.
- When ϵ→∞: Noise amplitude approaches zero, and clustering performance reaches the upper bound. However, differential privacy protection fails completely, and adversaries can infer raw features from uploaded core points.
- When ϵ=1: It achieves a balanced equilibrium. As validated by our parameter sensitivity experiments (Fig 4), ϵ=1 strikes the optimal balance across all eight datasets. Mathematically, with Δf=1, moderate noise intensity under ϵ=1 effectively resists single-sample inference attacks without excessive distortion of data density structures.

We add targeted configuration rules for three typical application scenarios involved in our paper: For medical data clustering (Heart, Breast datasets, high privacy demand), we recommend ϵ∈[0.1,0.5]. Slight performance loss is acceptable to strictly prevent leakage of sensitive patient information. For general image recognition tasks (MNIST, USPS, Olivetti face dataset), the default ϵ=1 is suitable. It balances privacy security and clustering accuracy for image clustering, segmentation and face recognition. For public geographic resource datasets (Covertype, Abalone, low privacy risk), we suggest ϵ∈[5,10]. Less noise interference is introduced to maximize clustering performance with negligible privacy threats.

**Parameters:** FDBSCAN has only one parameter, *i.e.,* the number of global centers called *k*. In addition, in the process of privacy protection, the differential privacy method has a parameter privacy budget ϵ, but we set it to ϵ=1 for all the situations, which is a fixed value.

## 4. FDBSCAN++

Based on FDBSCAN, we propose a new FDBSCAN++. FDBSCAN has four steps, for FDBSCAN++, only the third step is different from FDBSCAN. For the third step, the server aggregates the core points from all the clients (*CP*), and we use k-means to obtain the final global centers (*center*) as follows:


$$\begin{array}{c} \text{center}=\text{k-means}(\text{CP},k), \end{array}$$ (12)

where *k* is a user-defined parameter.

The core points calculation of the client is based on encrypted data ($p_{i}$), and even if there are security risks at the server, the original data will not be leaked. Finally, the server sends the final global centers (*center*) to each client.

This step has only *O*(*n*) time. The time complexity of the third step of FDBSCAN++ is one order of magnitude lower than that of the third step of FDBSCAN.

Classical improved DBSCAN baselines (HDBSCAN, aNNE-DBSCAN, MDBSCAN, etc.) adopt centralized computing frameworks, which require all raw data to be collected on a single machine. For large-scale datasets including MNIST and Covertype (over 580,000 samples), global neighborhood searching and pairwise distance calculation bring quadratic computational and memory complexity, inevitably leading to memory overflow (marked as NaN in our experimental tables).

In contrast, our federated framework partitions raw data across multiple clients. Each client executes lightweight adaptive DBSCAN on local differentially private data and only uploads compact core points rather than complete raw samples. The server merely aggregates a small set of core points, which drastically reduces computation and storage overhead. This structural design theoretically guarantees stable clustering on massive datasets, an advantage that centralized DBSCAN variants cannot possess.

Standard DBSCAN and its improved variants rely heavily on manual tuning of *Eps* and *minPts*; inappropriate parameter settings severely degrade clustering performance. Our adaptive DBSCAN generates pseudo-labels via K-means and iteratively optimizes *Eps* and *minPts* by maximizing the Adjusted Rand Index (ARI) without human intervention.

Theoretically, ARI quantitatively measures the consistency between density clustering outputs and the intrinsic category distribution of raw data. Optimizing ARI guarantees that extracted core points accurately reflect the true local density structure, laying a high-quality foundation for subsequent global aggregation. Existing federated clustering baselines (k-FED, MUFC, SFK) only adopt partition-based K-means locally and ignore density features, making them incapable of capturing arbitrarily shaped clusters and leading to inherent performance gaps.

**FDBSCAN**: Global cluster centers are selected via $\gamma_{i}=\rho_{i}\delta_{i}$, where local density $\rho_{i}$ and relative distance $\delta_{i}$ are computed based on shared nearest neighbor (SNN) structural similarity. This density-aware center selection conforms to the core logic of DBSCAN and naturally adapts to datasets with uneven density distribution, while federated K-means variants lack density perception capability.

**FDBSCAN++**: The *O*(*n*^2^) SNN-based density screening is replaced by lightweight K-means aggregation over uploaded core points, reducing the server-side time complexity to *O*(*n*). Meanwhile, core points uploaded from clients retain complete local density information. Other federated clustering algorithms aggregate ordinary local centroids without filtering noise and border points, which disturbs global center selection and deteriorates final clustering accuracy.

## 5. Experiments and discussions

In this section, we use extensive datasets to comprehensively evaluate the proposed FDBSCAN and FDBSCAN++ with five real-world UCI datasets: Iris, Seeds, Heart, Abalone, and Breast, and they are small datasets. We also use three large-scale datasets: USPS, Covertype, and MNIST, and we use the UMAP algorithm to reduce them to two dimensions. The features of all the datasets used in this paper are shown in Table 1.

**Table 1:** Dataset features.

ID	Datasets	#Samples	#Dimensions	#Natural clusters
1	Iris	150	4	3
2	Seeds	210	7	3
3	Heart	303	13	2
4	Abalone	4,177	8	3
5	Breast	277	9	2
6	USPS	11,000	256	10
7	Covertype	581012	54	7
8	MNIST	70,000	784	10

In addition, we also apply the FDBSCAN++ to image clustering and segmentation tasks, which achieves satisfactory results.

The datasets used in this paper are not federated datasets, we change traditional datasets into federated datasets using the method described in the paper [8], which is a popular way as shown in Algorithm 3. In addition, we set the number of clients num_clients=10.

**Algorithm 3: The details of generating federated datasets**



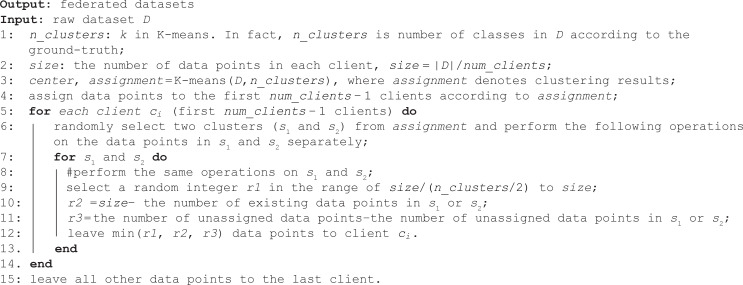



### 5.1. Benchmarking models

We compare FDBSCAN and FDBSCAN++ with six DBSCAN-related benchmarking models: DBSCAN [1], HDBSCAN [19], aNNE-DBSCAN (abbreviated as aNNE) [20], AMD-DBSCAN (abbreviated as AMD) [21], ST-DBSCAN (abbreviated as ST) [22], and MDBSCAN [23].

- **DBSCAN** was published in KDD 1996, it is a well-known density-based clustering algorithm.
- **HDBSCAN** and **ST-DBSCAN** are classical improved DBSCAN;
- **aNNE-DBSCAN** was published in AAAI 2019;
- **AMD-DBSCAN** was published in DSAA 2022;
- **MDBSCAN** was published in 2024.

The first three are classic algorithms, and the last two are SOTA DBSCAN extensions.

The federated clustering is an emerging field, and open-sourced code is very rare. In this research, we compare the proposed methods with three well-known algorithms: k-FED [24], MUFC [25] and SFK [8]:

- **k-FED.** A SOTA method of federated clustering algorithm published in ICML 2021 [24]. Its parameters are *k* (*k* of k-means at the server) and *kprime* (the number of uploaded centers);
- **MUFC.** Another SOTA method of federated clustering algorithm published in ICLR 2023 [25]. Its parameters are *k* (*k* of k-means at the server) and *kprime* (the number of uploaded centers);
- **SFK.** It is a SOTA federated k-means clustering algorithm published in TNNLS 2025 [8], it has three parameters: (client_k/k/server_k).

Many papers improved DBSCAN or use DBSCAN to solve some problems, but there are very few papers involving open-source codes, we try our best to search for an improved DBSCAN with codes (MATLAB or PYTHON). The code of our method can be downloaded from https://github.com/DZLONG0712/FDBSCAN, and anyone can download data from https://github.com/mlyizhang/Clustering-Datasets.

### 5.2. Evaluation Index

We use two common evaluation metrics, ARI and NMI, to evaluate all clustering results. For these two evaluation metrics, the higher the value, the higher the performance of the clustering algorithm. We test all algorithms using a unified hardware environment, i7-10750H CPU, 16.0GB RAM, and 64-bit Windows operating system, and we use PyCharm 2025 to implement FDBSCAN.

### 5.3. Comparing with DBSCAN-related clustering algorithms

In this paper, we compare FDBSCAN and FDBSCAN++ with previous baselines, including DBSCAN, HDBSCAN, aNNE, AMD, ST, and MDBSCAN. See Table 2, for eight real-world datasets, FDBSCAN++ achieves the best clustering results based on ARI and NMI scores and FDBSCAN obtain the optimal results in most cases. Especially for large-scale datasets USPS, Covertype, and MNIST, FDBSCAN and FDBSCAN++ achieve the highest scores, other improved DBSCAN methods aNNE, AMD, ST, and MDBSCAN do not obtain clustering results because they have high time complexity and have a memory explosion issue in our hardware environment (16.0 GB RAM). It can be seen that FDBSCAN and FDBSCAN++ have unique advantages in large-scale dataset clustering tasks, which is also the benefit brought by the distributed computing of the federated clustering framework.

**Table 2:** Comparison Between the proposed two methods and DBSCAN-related Work on Eight Datasets. Parameter values are in parentheses, the best clustering results are highlighted in bold, the suboptimal results are underlined and NaN denotes out of memory.

	Iris		Seeds		Heart		Abalone	
	ARI	NMI	ARI	NMI	ARI	NMI	ARI	NMI
DBSCAN	0.71(0.4/3)	0.71(0.4/3)	0.46(0.7/2)	0.51(0.7/2)	0.08(0.7/2)	0.14(0.7/2)	0.04(0.2/2)	0.09(0.2/2)
HDBSCAN	0.57(3/3)	0.73(3/3)	0.37(5/2)	0.49(5/2)	0.03(3/2)	0.13(3/2)	0.04(3/2)	0.10(3/2)
aNNE	0.57(0.7/3)	0.73(0.7/3)	0.75(0.4/2)	0.69(0.4/2)	0.06(0.4/3)	0.15(0.4/3)	0.12(0.1/5)	0.14(0.1/5)
AMD	0.56(/)	0.71(/)	0.31(/)	0.39(/)	0.05(/)	0.11(/)	0.07(/)	0.11(/)
ST	0.57(1.5/3)	0.73(1.5/3)	0.26(0.4/2)	0.43(0.4/2)	0.07(1/3)	0.11(1/3)	0.04(0.5/3)	0.09(0.5/3)
MDBSCAN	0.84(4/1.3 /0.4/4)	0.84(4/1.3 /0.4/4)	0.43(5/0.8 /0.9/5)	0.51(5/0.8 /0.9/5)	0.08(3/1/ 0.9/5)	0.11(3/1/ 0.9/5)	0.04(3/2/ 0.9/5)	0.09(3/2/ 0.9/5)
**FDBSCAN**	0.76(3)	0.77(3)	0.70(3)	0.67(3)	0.16(2)	0.15(2)	0.14(3)	0.14(3)
**FDBSCAN ++**	**0.90**(3)	**0.89**(3)	**0.76**(3)	**0.74**(3)	**0.40**(2)	**0.31**(2)	**0.16**(3)	**0.15**(3)
	**Breast**		**USPS**		**Covertype**		**MNIST**	
	**ARI**	**NMI**	**ARI**	**NMI**	**ARI**	**NMI**	**ARI**	**NMI**
DBSCAN	0.17(2.1/2)	0.08(2.1/2)	0.56(0.1/2)	0.68(0.1/2)	0.00(0.1/4)	0.03(0.1/4)	0.66(0.1/2)	0.82(0.1/2)
HDBSCAN	0.01(5/3)	0.03(5/3)	0.35(5/3)	0.54(5/3)	0.02(1/3)	0.09 (1/3)	0.72(2/2)	0.76(2/2)
aNNE	0.02(0.1/2)	0.09(0.1/2)	0.02(0.1/5)	0.15(0.1/5)	NaN	NaN	NaN	NaN
AMD	0.07(/)	0.11(/)	0.47(/)	0.67(/)	NaN	NaN	NaN	NaN
ST	0.02(0.5/5)	0.02(0.5/5)	0.00(0.05/5)	0.04(0.05/5)	NaN	NaN	NaN	NaN
MDBSCAN	0.01(5/5/ 1/2)	0.00(5/5/ 1/2)	0.00(5/4/ 7/3)	0.01(5/4/ 7/3)	NaN	NaN	NaN	NaN
**FDBSCAN**	0.16(3)	0.13(3)	0.60(10)	0.75(10)	0.03(7)	0.13(7)	0.87(10)	0.86(10)
**FDBSCAN ++**	**0.19**(2)	**0.11**(2)	**0.68**(10)	**0.76**(10)	**0.04**(7)	**0.13**(7)	**0.90**(10)	**0.90**(10)

### 5.4. Comparing with SOTA federated clustering algorithms

In this section, we compare the proposed methods with three SOTA federated clustering algorithms: k-FED, MUFC, and SFK. As shown in Table 3, for all the used datasets, the two proposed algorithms perform better than k-FED, MUFC, and SFK with a very significant advantage in most cases.

**Table 3:** Comparison Between the proposed two methods and SOTA federated clustering on Eight Datasets. Parameter values are in parentheses, the best clustering results are highlighted in bold, and the suboptimal results are underlined.

	Iris		Seeds		Heart		Abalone	
	ARI	NMI	ARI	NMI	ARI	NMI	ARI	NMI
k-FED	0.73(4/1)	0.75(4/1)	0.70(3/2)	0.69(3/2)	0.28(2/2)	0.21(2/2)	0.15(2/1)	0.15(2/1)
MUFC	0.73(3/2)	0.77(3/2)	0.69(3/3)	0.69(3/3)	0.18(2/2)	0.15(2/2)	0.13(3/2)	0.13(3/2)
SFK	0.81(10/3)	0.88(10/3)	0.75(10/3)	0.73(10/3)	0.38(10/2)	0.30(10/2)	**0.17**(10/3)	0.15(10/3)
**FDBSCAN**	0.76(3)	0.77(3)	0.70(3)	0.67(3)	0.16(2)	0.15(2)	0.14(3)	0.14(3)
***FDBSCAN ++***	**0.90**(3)	**0.89**(3)	**0.76**(3)	**0.74**(3)	**0.40**(2)	**0.31**(2)	0.16(3)	**0.15**(3)
	**Breast**		**USPS**		**Covertype**		**MNIST**	
	**ARI**	**NMI**	**ARI**	**NMI**	**ARI**	**NMI**	**ARI**	**NMI**
k-FED	0.16(2/1)	0.08(2/1)	0.60(10/2)	0.73(10/2)	0.01(10/10)	0.07(10/10)	0.75(10/2)	0.84(10/2)
MUFC	0.17(2/1)	0.08(2/1)	0.63(10/2)	0.75(10/2)	0.00(3/3)	0.07(10/12)	0.77(10/2)	0.85(10/2)
SFK	0.17(10/3)	0.11(10/3)	0.60(150/10)	0.73(150/10)	0.00(10/5)	0.08(10/5)	0.77(200/10)	0.85(200/10)
**FDBSCAN**	0.16(3)	0.13(3)	0.60(10)	0.75(10)	0.03(7)	0.13(7)	0.87(10)	0.86(10)
***FDBSCAN ++***	**0.19**(2)	**0.11**(2)	**0.68**(10)	**0.76**(10)	**0.04**(7)	**0.13**(7)	**0.90**(10)	**0.90**(10)

Given the stochastic nature of clustering and federated setups, repeated experiments and robustness analysis are essential to validate the claimed improvements, we make 3000 times experiments and report the clustering results with mean ± standard deviation as shown in Table 4. Compared with existing methods, the proposed method still has an advantage.

**Table 4:** Clustering results of 3000 experiments.

Datasets	ARI	NMI
Iris	0.92±0.01	0.91±0.01
Seeds	0.77±0.01	0.75±0.01
Heart	0.39±0.02	0.33±0.02
Abalone	0.17±0.01	0.33±0.02
Breast	0.21±0.01	0.15±0.02
USPS	0.68±0.01	0.78±0.01
Covertype	0.03±0.01	0.12±0.02
MNIST	0.89±0.02	0.90±0.01

### 5.5. Parameter sensitivity experiment for FDBSCAN++

**Fig 2:**
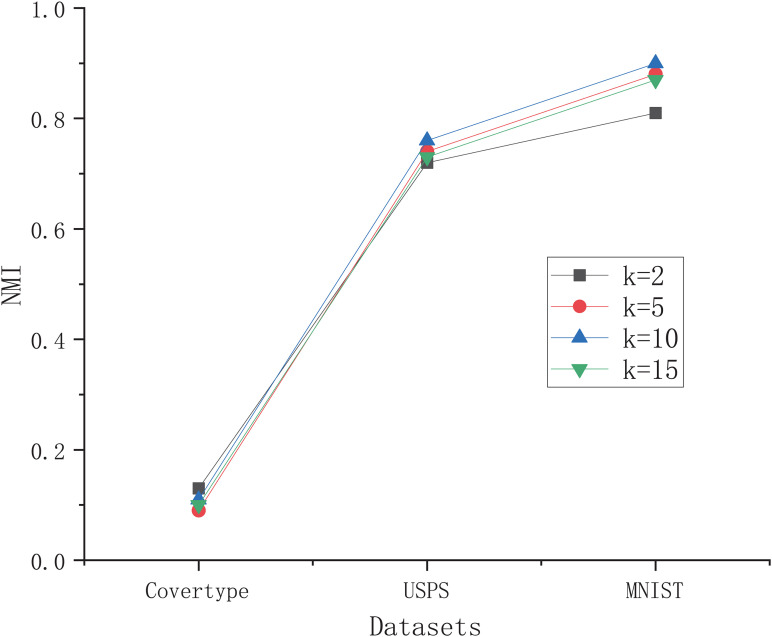
The influence of parameter *k* on clustering results using FDBSCAN++.

To illustrate the impact of various parameters on the clustering results of FDBSCAN++, we conduct a large number of parameter sensitivity experiments. FDBSCAN++ has one parameter *k* (the number of global centers, *i.e.,* the number of final clusters). In addition, during the experiments, we use fixed values for two important variables. However, we still conduct sensitivity tests to demonstrate their impact on the clustering results: the number of clients num_clients and the privacy budget ϵ. The specific experimental results are as follows:

**Parameter *k:*** The influence of parameters *k* on clustering results is shown in Fig 2. According to the experimental results, there is no clear pattern in the influence between parameters and clustering results.

**Fig 3:**
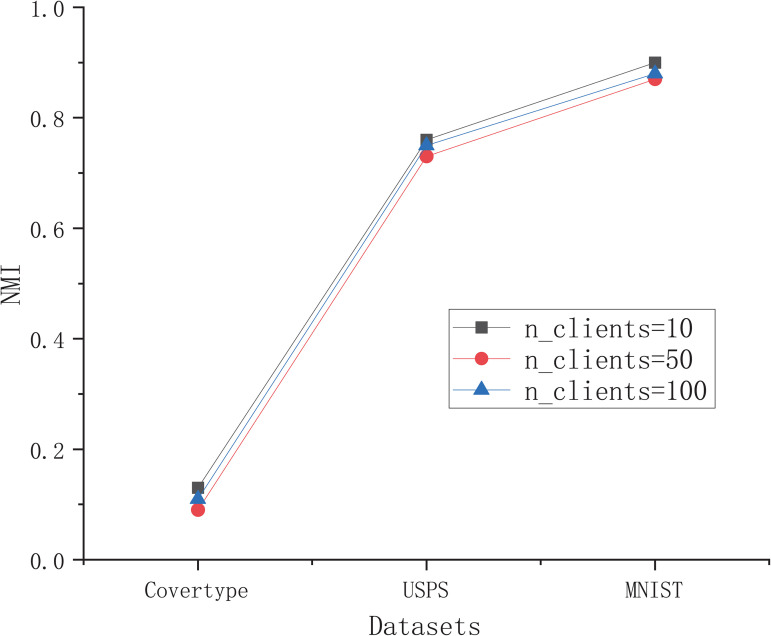
The influence of num_clients on clustering results using FDBSCAN++.

**The number of clients** num_clients: The influence of num_clients on clustering results is shown in Fig 3. According to the experimental results, there is no clear pattern in the influence between the number of clients and clustering results.

**Fig 4:**
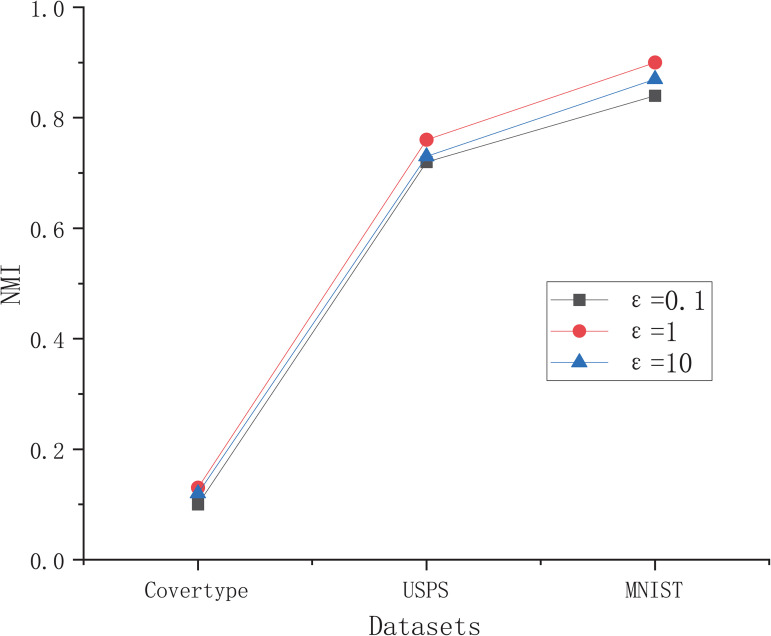
The influence of ϵ on clustering results using FDBSCAN++.

**Privacy budget** ϵ: The influence of ϵ on clustering results is shown in Fig 4. According to the experimental results, there is no clear pattern in the influence between privacy budget and clustering results.

Based on the sensitivity analysis of the above parameters, the settings of these parameters should be continuously adjusted according to the actual situation. The number of clients depends on how many terminals participate in the federated clustering system, and the privacy budget is generally fixed. And the parameter *k* can be continuously adjusted based on the final clustering results.

### 5.6. Analysis of communication costs

FDBSCAN and FDBSCAN++ just need one round of communication. In addition, the communication information between the client and server sides is cluster centers, and the communication volume is much smaller than the client data. The complexity of communication time is *O*(*n*).

### 5.7. Statistical significance test results

We adopt the Wilcoxon signed-rank one-tailed test to conduct statistical significance analysis on ARI and NMI metrics. As shown in Table 5, all obtained P-values are less than 0.05, which fully verifies that the proposed FDBSCAN++ achieves statistically significant performance improvements compared with all traditional clustering algorithms and state-of-the-art federated clustering methods. Most P-values are lower than 0.01, further demonstrating that the superiority of FDBSCAN++ is extremely remarkable and stable across different evaluation indicators.

**Table 5:** Statistical significance test results between FDBSCAN++ and comparison methods.

Algorithm	P-value(ARI)	P-value(NMI)
DBSCAN	0.00	0.00
HDBSCAN	0.00	0.00
aNNE	0.02	0.05
AMD	0.02	0.02
ST	0.02	0.02
MDBSCAN	0.03	0.03
k-FED	0.02	0.01
MUFC	0.01	0.01
SFK	0.04	0.04
FDBSCAN	0.00	0.00

### 5.8. Application of FDBSCAN++ in facial recognition

We apply FDBSCAN++ to the face clustering task on the Olivetti face dataset. As shown in Table 6, FDBSCAN++ achieved good clustering results, accurately grouping the faces of five people into one cluster.

**Table 6:** The clustering results of the Olivetti face dataset.

ID	ARI	NMI
DBSCAN	0.62	0.72
FDBSCAN	0.69	0.75
FDBSCAN++	**0.81**	**0.79**

### 5.9. Application of FDBSCAN++ in image segmentation

**Fig 5:**
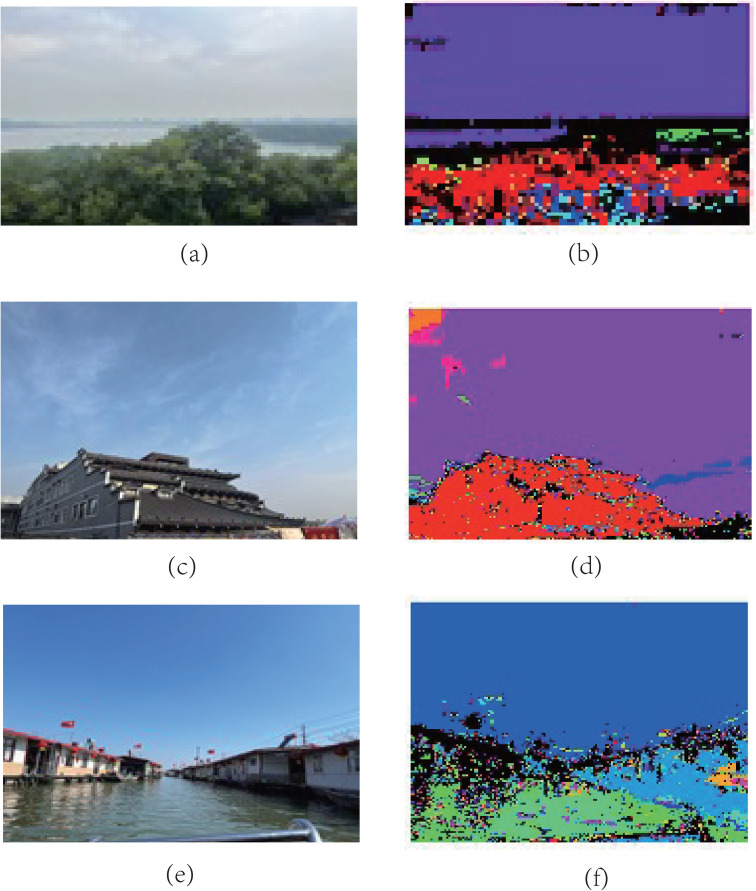
Original images and their segmentation results using FDBSCAN++. Note that all images are taken by the authors themselves and do not require permission from any individual or organization for use.

We apply FDBSCAN++ to the image segmentation task on four images: deer, lake, flower, and land. As shown in Fig 5, FDBSCAN++ achieved good results, accurately dividing the image into different parts based on semantic features, and the semantics of each part are relatively complete.

## 6. Conclusion and future work

In this paper, we propose two new DBSCAN clustering algorithms combining privacy protection and distributed computing based on a federated clustering framework, called FDBSCAN and FDBSCAN++. In the proposed methods, we also propose an adaptive DBSCAN clustering algorithm that utilizes other clustering algorithms to construct pseudo labels, helping DBSCAN find better parameter values. For traditional DBSCAN and its improved algorithms, large-scale data clustering has always been a challenge. For example, traditional improved DBSCAN algorithms struggle to handle MNIST datasets or data volumes of over 100,000. Our proposed FDBSCAN algorithm can easily cluster large-scale datasets (with Covertype containing 580,000 data points). We combine federated clustering with DBSCAN for the first time to achieve the above results.

In the future, we will conduct in-depth research on feature learning algorithms based on large language models, using language models to assist in learning the features of large-scale complex images, and establish a more universal federated density clustering algorithm.
